# Rett syndrome: think outside the (skull) box

**DOI:** 10.12703/r/10-59

**Published:** 2021-06-29

**Authors:** Emilie Borloz, Laurent Villard, Jean-Christophe Roux

**Affiliations:** 1 Aix Marseille Univ, INSERM, MMG, U1251, Faculté de médecine Timone, 13385, Marseille, France

**Keywords:** Rett syndrome, Mecp2, comorbidities, peripheral systems

## Abstract

Rett syndrome (RTT) is a severe X-linked neurodevelopmental disorder characterized by neurodevelopmental regression between 6 and 18 months of life and associated with multi-system comorbidities. Caused mainly by pathogenic variants in the *MECP2* (methyl CpG binding protein 2) gene, it is the second leading genetic cause of intellectual disability in girls after Down syndrome. RTT affects not only neurological function but also a wide array of non-neurological organs. RTT-related disorders involve abnormalities of the respiratory, cardiovascular, digestive, metabolic, skeletal, endocrine, muscular, and urinary systems and immune response. Here, we review the different aspects of RTT affecting the main peripheral groups of organs and sometimes occurring independently of nervous system defects.

## Introduction

Rett syndrome (RTT) is a severe X-linked neurological disorder that is caused primarily by loss-of-function mutations in the ubiquitously expressed *MECP2* (methyl CpG binding protein 2) gene^1^. It is the second most prevalent genetic cause of intellectual disability in girls^2^ (incidence of 1/10,000 girls) after Down syndrome. Mutations occur *de novo*, preventing the possibility of prenatal screening or genetic counseling; therefore, the incidence is not expected to decline. After a period of normal development, patients with RTT undergo a regression of early neurodevelopment^3^. The main clinical features are severe intellectual disability, microcephaly, loss of hand skills and speech, seizures, and respiratory and motor abnormalities^4^.

Although neurological conditions are prominent, the disease affects not only the central nervous system (CNS) but also a wide array of non-neurological organs. RTT has a complex and multifaceted clinical appearance^5^. This pathology evolves throughout the life span of the patient, and multi-system comorbidities, like gastrointestinal (GI), orthopedic, endocrine, or cardiac issues, are more or less prevalent^6^. Liver injury, urological dysfunction, adipose tissue disorders, and inflammatory response troubles are also non-neurological disorders associated with RTT^7^. Although most research is focused on the role of *MECP2* in the CNS, the different clinical aspects identified in *Mecp2* mutant animal models and RTT patients underline the importance of MECP2 in peripheral tissues. Even if most symptoms of RTT arise from neural origin, a mouse model exclusively expressing Mecp2 in neuronal and glial cells suggests that some symptoms of RTT occur independently of nervous system defects^8^.

According to caregivers, many of these chronic health issues cause pain and impair the quality of life of patients with RTT^9^. Currently, treatments are aimed at alleviating symptoms but not cure RTT^10,11^ and these peripheral abnormalities should be considered when planning therapeutic strategies. Here, we review the main systems affected by *MECP2* mutations, besides the CNS, in RTT patients and mouse models.

## Respiratory system

The most prevalent peripheral dysfunctions in RTT are breathing abnormalities^12^. Nearly 100% of patients with classic RTT will develop, over their life span, breathing abnormalities that can be categorized into one of two groups: hyperventilation and breath-holding^13^. Up to a quarter of sudden RTT-associated deaths could be caused by respiratory arrhythmia^14^. Both male and female *Mecp2*-deficient mouse models (hemizygous or heterozygous *Mecp2*-knockout (KO) mice) display breathing disturbances with apneas, beginning around 1 month of life and worsening over time, together with an exacerbated response to hypoxia^15–18^. Post-mortem analysis of *Mecp2*-null mice lungs showed macroscopic and histological abnormalities with the presence of infiltrating cells in different tissues of the lungs, indicating inflammation^19^.

The cause of these breathing irregularities has been investigated in RTT mouse models, principally in the brain stem, which has a critical role in regulating respiratory function. A conditional *Mecp2*-KO in the brain stem and spinal cord caused abnormal patterns of breathing^20^. Many neuronal mechanisms are likely to play a role in the RTT breathing phenotype^21^ as imbalances in synaptic transmission^22^ and alteration in different neuromodulatory systems such as bioaminergic neurotransmission^15,18^.

A peripheral contribution to the respiratory symptoms has nevertheless been examined. Although Mecp2 is expressed in the lungs, its conditional deletion in peripheral tissues is not sufficient to perturb breathing activity. Unlike in the Mecp2-null mice, in conditional peripheral Mecp2-KO mice no gross structural abnormalities or inflammation were found in lung tissue biopsies^8^. However, the presence of Mecp2 is essential for pulmonary fibrosis^23^. Together, these results confirm that respiratory dysfunction is linked to disruption of autonomic or brain stem function.

## Digestive system

GI and nutritional troubles are frequently reported in patients with RTT^24^. In a cohort of 983 females with RTT, 81% had feeding problems (principally chewing difficulty, prolonged feeding time, and choking), 92% had GI problems (gastroesophageal reflux, constipation, straining with bowel movements, and passage of hard stools), and 47% had nutritional problems (poor weight principally)^24^. A small percentage (4.4%) of patients also have biliary tract disease, which may have a fatal outcome^25^.

Only a few studies have investigated GI dysfunction in mouse models and, although there is a well-known underweight in *Mecp2*-KO mice^26^, GI dysmotility was described only in 2016^27^. That study found a large increase in transit time in *Mecp2*-KO mice compared with controls^27^. Wahba *et al*. also showed that MECP2 was expressed in the GI tract, specifically in the enteric nervous system (ENS) in humans and mice as early as embryonic day 11.5 (E11.5)^28^. GI motility is controlled by the myenteric plexus, part of the ENS which uses more than 30 neurotransmitters^29^. However, peristalsis is activated mainly by acetylcholine and inhibited by vasoactive intestinal peptide^29^ and nitric oxide^30^. Levels of nNOS (the enzyme catalyzing the production of nitric oxide) were elevated in *Mecp2*-KO GI tissue^27^. This upregulation could be, in part, responsible for GI hypomotility.

At the histological scale, *Mecp2*-null mice displayed a shorter colon with severe changes in its epithelium similar to those observed in colitis and abnormal localization of key membrane proteins^31^. However, conditional deletion of *Mecp2* from intestinal tissue using the villin promoter, an actin binding protein expressed mainly in the microvilli of the epithelium of the gut, does not reproduce the *Mecp2*-null GI phenotype^31^. Since the conditional Mecp2-KO in non-neuronal cells does not display any body-weight decrease compared with the control group^8^, these symptoms may have a neural origin. A central or enteric origin cannot be ruled out here since a brain- or ENS-specific *Mecp2*-KO does not exist. The commonly called CNS conditional KO mice were generated with the nestin-Cre transgene^26,32^. However, nestin is also expressed in the gut, and nestin-expressing intestinal cells give rise to enteric neurons and glia^33^. A study by Deguchi *et al*. suggests that constipation in RTT is related to autonomic nervous system dysfunction originating in the brain stem and not in the bowel^34^. Indeed, an immunoreactivity assay showed no differences in the expression of substance P, tyrosine hydroxylase, and vasoactive intestinal peptide in girls with RTT compared with healthy controls in the gut. This is in contrast to the brain stem, where differences were found^34^. However, GI motility is not controlled by these neurotransmitters only, and transit perturbations could be partly caused by ENS dysfunction.

Prompted by recent evidence that bacterial and fungal gut microbiota have a role in human health and GI functions, studies have been conducted in patients with RTT^35–37^. Measures of fecal calprotectin and erythrocyte sedimentation rate showed an increase linked to intestinal sub-inflammatory status in patients with RTT^36^. Interestingly, patients with RTT display an altered microbiota, in terms of abundances and richness, and modified short-chain fatty acid profiles^36^ and these alternations were not correlated with constipation status. The study by Borghi *et al*. (2017) was in agreement with the latest findings; however, the authors showed that microbiota dysbiosis was related to total disease severity^35^. A focus on the gut mycobiota revealed a distinct genotypic profile of *Candida parapsilosis* in girls with RTT compared with controls, increasing its potential virulent features and the capacity to be more resistant to antifungals^37^. Even though mechanisms are not clearly understood yet, altered gut microbiota could be implicated in the GI pathophysiology of RTT. To resolve this question, intestinal microbiota was investigated in a female rat model of RTT^38^, and different abundance of microbial taxa between wild-type (WT) and RTT rat has been identified. The use of animal models allows experimental control of the diet and the host, and manipulation of the microbiota is necessary to further investigate the role of the gut microbiome in the severity and progression of RTT.

## Metabolism

The liver plays a central role in all of the body’s metabolic functions, such as carbohydrate, fat, or protein metabolism^39^. Interestingly, RTT is associated with metabolic dysfunctions and liver disease^40^.

Kyle *et al*. (2016) investigated whether the loss of *Mecp2* could cause perturbations of metabolism in a male mouse model of RTT^41^. *Mecp2* deletion induces severe dyslipidemia, fatty liver disease, metabolic syndrome, and insulin resistance and alters energy homeostasis. Liver-specific deletion of *Mecp2* increases lipogenic enzyme transcription, leading to fatty liver disease without affecting insulin sensitivity^41^.

Except for the brain, which has to synthesize its own cholesterol (since this compound cannot cross the blood–brain barrier), the body’s primary producer of cholesterol is the liver^42^. Although brain cholesterol synthesis is commonly disrupted in *Mecp2*-null mice, elevated cholesterol level in the liver is observed only in 129.Mecp2^tm1.1Bird^ mice^43^, and peripheral metabolic phenotype differs across genetic backgrounds^44^. Consistently, lipid metabolism is altered in a subset of patients with RTT^45^. The imbalance in plasma lipid profile is restricted to cholesterol metabolism with augmented levels of total cholesterol, low-density lipoprotein (LDL) and high-density lipoprotein (HDL) cholesterol in patients with RTT^46^.****

The understanding of metabolic aspects and liver injuries linked to RTT reveals potential therapeutic targets but unfortunately faces limitations for therapeutic strategies such as gene therapy. Indeed, hepatotoxicity due to high off-target liver delivery of an AAV9-Mecp2 has been demonstrated in a female mouse model of RTT^47^.

## Skeletal system

Orthopedic comorbidities have been reported in over 80% of patients with RTT, and the most encountered disorder is scoliosis^48^. Scoliosis is related to a lack of walking but is unrelated to the loss of hand skills or hand stereotypes^48^. These results extend our understanding of interactions between scoliosis and overall clinical severity. Surgical correction of scoliosis is mostly successful but has a very high rate of complications with a prolonged hospital stay^49^. Other orthopedic symptoms include hip displacement and a high prevalence of fractures^50–52^, which can be diminished with intravenous bisphosphonates treatment^53^. Several markers, such as osteocalcin or bone-specific alkaline phosphatase, which are linked to bone formation and resorption, were decreased in patients with RTT^54^. These results suggest a low bone turnover that could explain altered bone mass. Altogether, these observations support the need for clinical and radiological surveillance in all patients with RTT.

Studies in a murine model of RTT confirmed low bone mass as a component of this syndrome with cortical thickness, mineralization of the medullary cavity in long bones, and low bone density in the spine^55^. Curvature of the spine is also present with kyphosis in the form of a C^56^. Interestingly, Kamal *et al*. (2015) showed that the skeletal phenotype caused by *Mecp2* deficiency is potentially reversible by the delayed restoration of *Mecp2* in adult mice^57^. These results highlight the importance of the development of gene-based therapies, especially in light of recent advances in gene therapy for bone regeneration^58^.

## Endocrine system

The endocrine system is controlled by glands, located either in the brain (such as the pituitary and the pineal gland) or in the periphery (such as the thyroid, adrenals, or gonads). Other tissues, like the adipose tissue, which secretes leptin, a hormone inhibiting hunger and upregulated in RTT, can also contribute to the endocrine system^59^. Endocrine disorders have an impact on growth, menstrual cycles, and bones.

Hyperactivity of the hypothalamic–pituitary–adrenal (HPA) axis has been observed in animal models of RTT^60^, and the investigation of HPA axis function among patients with RTT shows that diurnal decline of cortisol is less steep in patients with the most severe symptoms such as frequent hyperventilation^61^. These preliminary results support the hypothesis that RTT is associated with aberrant HPA axis function.

In patients with RTT, the most common (59% of patients) endocrine issue is low bone mineral content^52^, which is correlated with the occurrence of fractures. This alteration of bone mineral deposition may be caused by vitamin D deficiency^62^. Blue *et al*. (2015) showed that, compared with WT mice, female heterozygous and male *Mecp2*-null mice also displayed decreased mineral apposition rate, mineralizing surface, and bone formation rate/bone surface^63^.

Girls with RTT also frequently present alterations in the time of appearance of secondary sexual characteristics such as early pubertal onset and delayed menarche^64^, yet the mechanisms by which *MECP2* mutations could impact pubertal pathways remain unclear.

Also, at the gland level, thyroid function has been studied in RTT and no consensus was reached^65–67^. A subtle decrease of levels of T_4_, one of the thyroid hormones, was reported in a cohort of 17 patients by Cooke *et al*., but no evidence of clinical hypothyroidism was found^67^. The study of a larger cohort of 45 girls with typical and atypical RTT showed opposite results with increased T_4_ levels^65^. The thyroid hormones T_3_ and T_4_ are essential for proper development; they function as transcription factors and the genes they regulate are important for brain development^68^. The relationship between thyroid disorders and RTT phenotype merits further investigations, especially since it is known that the lack of thyroid hormones results in intellectual deficiency^68^ and weight variation^69^.

## Cardiovascular system

Cardiac abnormalities have been investigated as a cause of sudden death of unknown origin in RTT. The patients have electrocardiogram and rhythm defects, including prolonged QT interval (QTc)^70,71^, one of the most studied cardiac risk factors for sudden death. No clinical morphological or functional changes have been demonstrated using cardiac imaging studies, although a subclinical mild decrease in systolic and diastolic ventricular function was found in a cohort of 72 girls with typical RTT^72^. These findings must be considered in the context of patients exhibiting autonomic dysfunctions with sympathetic over-activity, parasympathetic under-activity, and sympathovagal imbalance^73^.

Cardiac arrhythmias have also been reported in animal models of RTT; and these arrhythmias could be predisposed by prolonged QTc^74^ but also by the abnormal differentiation of cardiac progenitors, the dysregulation of cardiac genes, or cardiomyocyte structural alterations^75^. Baseline heart rate and blood pressure are at WT levels in *Mecp2* heterozygous females^76^, suggesting no defects of autonomic cardiovascular control. However, Herrera *et al*. (2016) showed that conditional removal of *Mecp2* in cholinergic neurons was sufficient to recapitulate cardiac rhythm defects in RTT mice^77^. These results showed that *Mecp2* deficiency altered autonomic cardiac control mainly via the cholinergic nervous system.

## Muscular system

Muscular troubles are present at different time points of the pathology. Mild hypotonia is frequently observed before the onset of symptoms in patients with RTT^78^. During the late motor deterioration phase, abnormal muscle tone is observed^79^. Skeletal muscles in Mecp2 mutant mice were not extensively investigated except by Conti *et al*. (2015)^80^. Mecp2 deficiency induces severe skeletal muscle atrophy with no dystrophic features other than a reduced muscle mass^80^. Muscle atrophy can have different causes, including necrosis (which is not observed here), abnormal innervation, or dysfunction of the neuromuscular plaque. However, the morphology of neuromuscular plaques is normal in the absence of Mecp2^80^ and innervation is functional^81^. Another cause of atrophy could be the affected regeneration after muscular injuries observed in *Mecp2*-null mice^80^.

To understand whether muscular defects are cell- or non-cell-autonomous, Conti *et al*. generated a conditional KO for Mecp2 using the MyoD promoter, expressed in myoblasts and muscle fibers. These animals displayed normal muscle structure and myofibers and did not show any of the RTT-linked myopathy^80^. According to these results, Mecp2 is not required for the development and growth of skeletal muscles and these defects may be due to non-cell-autonomous mechanisms related to trophic factors^80^.

Skeletal muscles are rich in mitochondria, the primary organelle producing energy in the cell. Muscle biopsies of patients with RTT showed not only morphological ultrastructural abnormalities in mitochondrial number and size but also distention, vacuolation, dumb-bell shape, and membranous changes^82–86^. Therefore, the muscular phenotype of RTT could also be caused, in part, by mitochondria dysfunction.

## Urinary system

Urological dysfunction is infrequent but is described in a subset of patients, about 8% of a large cohort of 905 girls with typical RTT^87^, which is a higher incidence rate compared with the general population. The most frequent complications are urinary tract infection, kidney stones, or urine retention^87^ but also urinary acidification^88^. These symptoms are significantly correlated with the total clinical severity score. However, incontinence is not part of the RTT phenotype^89^.

In both male and female *Mecp2*-mutant mice, urological function was studied in the absence of Mecp2^87^. Patterns of micturition were assessed, and abnormalities were found with a decreased volume of urine per spot for both sexes; no difference was found between hemizygous males and heterozygous *Mecp2*-KO females. As micturition troubles can lead to urine reflux from the bladder to the kidney and cause damage, kidney function was also investigated. Serum chemistry exhibited evidence of kidney failure and necropsy identified distended bladders, obstruction of the urinary tract, and moderate hydronephrosis^87^; this clinical observation could be a cause of mortality in these mice.

The cause of urological dysfunction in RTT is not well known yet. Nevertheless, it is noteworthy that urological problems are found in other neurological disorders like Parkinson’s disease or Huntington’s chorea. Urological complications could be the consequence of a dysfunction of the autonomic nervous system.

## Immune response

A subclinical chronic inflammatory status has been shown in RTT patients with an increased erythrocyte sedimentation rate^90^. A major cytokine upregulation was also found and was correlated with clinical severity and the inflammatory status^91^. These observations suggest that immune response dysfunctions contribute to the pathophysiology of RTT^92^: on one hand, with the alteration of microglia activity in the CNS that affects neural development^93^ and, on the other hand, with the alteration of peripheral immune cells such as macrophages. Even if several reports suggest the presence of an autoimmune component in RTT, such as oxidative damage, cytokine dysregulation, or acute-phase protein response, no “classical autoimmunity” has been shown yet^94^.

Mecp2 was found to be expressed in cells implicated in the immune response, such as peripheral macrophages and monocytes^95^. In Mecp2-null mice, macrophage populations, including microglia, are decreased^96^. However, conditional KO of *Mecp2* in the macrophage was not able to generate an RTT phenotype, but the inflammatory status has not been reported yet^97^.

Immune response participates in RTT pathophysiology, and the genetic rescue of several macrophage and monocyte populations results in the attenuation of a subset of phenotypes and the increase of the life span^95^.

## Conclusions

**Figure 1.**
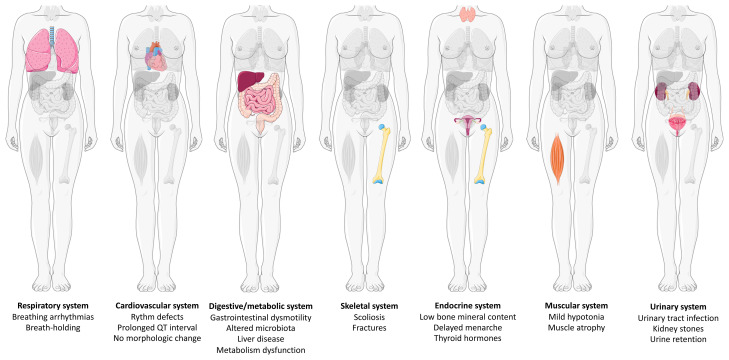
Rett syndrome-related organ system disorders involve abnormalities of the respiratory, cardiovascular, digestive, metabolic, skeletal, endocrine, muscular, and urinary system. Graphical representation of the organ systems affected in a patient with Rett syndrome. The schematic art pieces used in this figure were provided by Servier Medical Art (https://smart.servier.com/). Servier Medical Art by Servier is licensed under the terms of Creative Commons Attribution 3.0 Unported License (CC BY 3.0).

RTT is a complex disorder that impacts primarily the CNS but also several systems inducing multiple comorbidities (Figure 1). Most of these peripheral symptoms, such as breathing abnormalities or cardiac defects, are still due to Mecp2 deficiency in the brain or peripheral nervous system. However, some organs are likely affected by the lack of Mecp2 in the tissue itself. It is noteworthy that some comorbidities are also found in other diseases affecting multiple organs and are certainly consequences of other symptoms. Therefore, these clinical manifestations may not be specific to RTT.

The various studies on mouse models have shown that they reproduce certain comorbidities fairly accurately. However, these models, generally *Mecp2*-KO males, are genetically quite distant from the mutations found in female patients, complicating the translation of studies and therapeutic trials in patients.

In order to improve disease management and the patients’ quality of life, multi-disciplinary medical follow-up is key. Indeed, these “peripheral” symptoms cause significant pain and may be responsible for sudden death. The complexity of the disease is a hindrance to research, mainly for therapy, and the multi-organ damage is an additional challenge. A better understanding of this multi-system disorder, thanks to model systems and human studies, will provide better care for patients and hopefully advance therapeutic development.
